# Investigation of PET image quality with acquisition time/bed and enhancement of lesion quantification accuracy through deep progressive learning

**DOI:** 10.1186/s40658-023-00607-x

**Published:** 2024-01-10

**Authors:** Hongxing Yang, Shihao Chen, Ming Qi, Wen Chen, Qing Kong, Jianping Zhang, Shaoli Song

**Affiliations:** 1 Key Laboratory of Nuclear Physics and Ion-Beam Application (MOE), Fudan University, No. 220, Handan Road, Yangpu District, Shanghai, 200433 People’s Republic of China; 2 Department of Nuclear Medicine, Fudan University Shanghai Cancer Center, No. 270, Dong’an Road, Xuhui District, Shanghai, 200032 People’s Republic of China; 3 Department of Oncology, Shanghai Medical College, Fudan University, No. 130, Dong’an Road, Xuhui District, Shanghai, 200032 People’s Republic of China; 4 Center for Biomedical Imaging, Fudan University, No. 270, Dong’an Road, Shanghai, 200032 People’s Republic of China; 5 Shanghai Engineering Research Center for Molecular Imaging Probes, No. 270, Dong’an Road, Xuhui District, Shanghai, 200032 People’s Republic of China; 6 Institute of Modern Physics, Fudan University, No. 220, Handan Road, Yangpu District, Shanghai, 200433 People’s Republic of China; 7 Shanghai Key Laboratory of Bioactive Small Molecules, Fudan University, No. 220, Handan Road, Yangpu District, Shanghai, 200032 People’s Republic of China

**Keywords:** PET/CT, Deep progressive learning, Image quality, Sub-centimeter lesions quantification

## Abstract

**Objective:**

To improve the PET image quality by a deep progressive learning (DPL) reconstruction algorithm and evaluate the DPL performance in lesion quantification.

**Methods:**

We reconstructed PET images from 48 oncological patients using ordered subset expectation maximization (OSEM) and deep progressive learning (DPL) methods. The patients were enrolled into three overlapped studies: 11 patients for image quality assessment (study 1), 34 patients for sub-centimeter lesion quantification (study 2), and 28 patients for imaging of overweight or obese individuals (study 3). In study 1, we evaluated the image quality visually based on four criteria: overall score, image sharpness, image noise, and diagnostic confidence. We also measured the image quality quantitatively using the signal-to-background ratio (SBR), signal-to-noise ratio (SNR), contrast-to-background ratio (CBR), and contrast-to-noise ratio (CNR). To evaluate the performance of the DPL algorithm in quantifying lesions, we compared the maximum standardized uptake values (SUV_max_), SBR, CBR, SNR and CNR of 63 sub-centimeter lesions in study 2 and 44 lesions in study 3.

**Results:**

DPL produced better PET image quality than OSEM did based on the visual evaluation methods when the acquisition time was 0.5, 1.0 and 1.5 min/bed. However, no discernible differences were found between the two methods when the acquisition time was 2.0, 2.5 and 3.0 min/bed. Quantitative results showed that DPL had significantly higher values of SBR, CBR, SNR, and CNR than OSEM did for each acquisition time. For sub-centimeter lesion quantification, the SUV_max_, SBR, CBR, SNR, and CNR of DPL were significantly enhanced, compared with OSEM. Similarly, for lesion quantification in overweight and obese patients, DPL significantly increased these parameters compared with OSEM.

**Conclusion:**

The DPL algorithm dramatically enhanced the quality of PET images and enabled more accurate quantification of sub-centimeters lesions in patients and lesions in overweight or obese patients. This is particularly beneficial for overweight or obese patients who usually have lower image quality due to the increased attenuation.

## Introduction

Positron emission tomography/computed tomography (PET/CT) is widely used in oncology for tumor detection, staging and therapy response assessment. The quality of PET/CT images is crucial for the quantitative analysis of tumor metabolism, which is one of the advantages of PET/CT over conventional diagnostic modalities [1–3]. The PET reconstruction algorithm plays a key role in the accuracy of standardized uptake value (SUV) measurement [4–6], which reflects the tumor uptake of radiotracers. The most commonly used PET reconstruction algorithm is the ordered subset expectation maximization (OSEM) algorithm, which was proposed by Hudson in 1994 [7]. However, OSEM has some limitations, such as noise amplification and partial convergence, which affect the image quality and SUV accuracy. To overcome these drawbacks, post-smoothing methods are often applied to reduce noise, but they also lead to a loss of resolution and an underestimation of SUVs [8, 9].

In recent years, deep learning-based approaches have shown great potential in improving PET image quality and reducing noise [10–12]. Hu et al. [13] found that generative adversarial network (GAN) and convolutional neural network (CNN) could suppress image noise to varying degrees and improve image quality. Lu et al. [14] demonstrated that fully 3D U-net could effectively reduce image noise and control bias even for sub-centimeter small lung nodules when generating standard dose PET using 10% low count down-sampled data. Wang et al. [15] proposed a method of 3D conditional GANs which could achieve better performance than the state-of-the-art methods in both qualitative and quantitative aspects.

Deep progressive learning (DPL) is a novel CNN-based method that has been reported to have the ability to reduce noise and enhance the contrast of PET images [16, 17]. DPL algorithm employs two networks: a denoising network (CNN-DE) and an enhancement network (CNN-EH). CNN-DE can suppress the noise from the input image, while CNN-EH can map from a low convergent image to a high convergent image. The training images of DPL came from uEXPLORER [18], the world’s first clinical total-body PET scanner, in which the PET images showed high contrast and very low image noise.

In this study, we studied the quality of PET images versus acquisition time/bed through visual and quantitative analyses. Furthermore, we examined the performance of the DPL algorithm in the quantification of lesions in overweight and obese patients and sub-centimeter lesions in patients. Our results showed that the DPL algorithm can enhance the quality and accuracy of PET images significantly, especially for the quantification of sub-centimeter lesions in patients and lesions in overweight and obese patients.

## Methods

The details of DPL design, network training and testing have been reported in Ref. [16]. In this study, we are intended to assess the image quality and explore the potential clinical applications of the DPL algorithm. We evaluated the DPL algorithm in two aspects: visual and quantitative image analyses. For visual analysis, two physicians with more than 5 years of experience reviewed the maximum intensity projection (MIP) and transverse images of the PET series, which were sorted in a random order, using a dedicated reporting system. They were blind to the reconstruction method and the patient information. And then, they rated the overall image quality (overall score, image sharpness and diagnostic confidence) using a 5-point Likert scale (1. poor; 2. reasonable; 3. good; 4. very good; and 5. excellent quality) [9, 19]. The rating scale was reversed for image noise, where 5 meant poor and 1 meant excellent. For quantitative analysis, two physicians, who confirmed the data with each other, measured the SUVs of each lesion and the right lobe of the liver (parenchymal organ background). Three physicians delineated the volumes of interest (VOIs) with a 3.0 cm diameter sphere on different PET image slices of the liver. Only liver parenchyma with a normal appearance on both PET and CT was invoked as a reference. The mean SUV (SUV_mean_), the standard deviation of SUV (SUV_SD_) and the maximum SUV (SUV_max_) of the liver and the lesions within the VOIs were recorded for both OSEM and DPL reconstructions. The liver SUV_SD_ was utilized as a measure of noise. Based on these measurements, we calculated signal-to-background ratio (SBR), signal-to-noise ratio (SNR), contrast-to-background ratio (CBR) and contrast-to-noise ratio (CNR) as follows:

$$SBR = \frac{{SUV_{{{\text{max}}}} }}{{SUV_{{{\text{mean}}}} }},$$

$$SNR = \frac{{SUV_{{{\text{max}}}} }}{{SUV_{{{\text{SD}}}} }},$$

$$CBR = \frac{{SUV_{{{\text{max}}}} - SUV_{{{\text{mean}}}} }}{{SUV_{{{\text{mean}}}} }},$$

$$CNR = \frac{{SUV_{{{\text{max}}}} - SUV_{{{\text{mean}}}} }}{{SUV_{{{\text{SD}}}} }}.$$

## Patients

We enrolled 48 oncological patients (male/female: 21/15, age 37–77 years) who underwent clinical ^18^F-FDG PET/CT examinations at the Fudan University Shanghai Cancer Center (FUSCC) from July 2021to March 2023. Among them, 11 were used to evaluate the quality of reconstructed images using visual evaluation methods and quantitative numerical methods (study1), 34 were used to study the ability to quantify sub-centimeter lesions (study 2) and 28 were used to explore the ability to image overweight patients or obese patients (study3). For study 1, the main condition is the long acquiring time. The patients enrolled in study 1 should move free and have good ability to control themselves. For study 2, the main condition is sub-centimeter lesions. To make our results convincing, we have enrolled the 71% patients of the whole into study 2. For study 3, the main condition is BMI ≥ 24 kg/m^2^. Their clinical information is listed in Table 1. All patients had fasted for at least 6 h before the ^18^F-FDG injection, and their blood glucose levels were confirmed to be ≤ 10 mmol/L. The injection dose of ^18^F-FDG was based on the patient’s weight (3.7 MBq/kg). During an uptake period of about 60 min, the patients drank 500 mL of water. This study was approved by the FUSCC ethics committee and followed the FUSCC ethical standards, and all patients signed a written informed consent before the injection. In clinical practices, we defined a sub-centimeter lesion as having a maximum diameter ≤ 1 cm. According to the World Health Organization criteria, we classified the patients as overweight if their body mass index (BMI) was between 24 and 28 kg/m^2^, and as obese if their BMI was ≥ 28 kg/m^2^.

**Table 1:** Patient clinical characteristic

Parameters	Study 1	Study 2	Study 3
Age (years)	59 ± 7	59 ± 11	58 ± 11
	[41, 68]	[37, 77]	[36, 74]
*Gender*			
Male	7	20	15
Female	4	14	13
Weight (kg)	68.4 ± 13.5	72.0 ± 12.9	72.4 ± 10.3
	[45, 98]	[45, 110]	[60, 110]
Height (cm)	164 ± 6	165 ± 7	163 ± 7
	[156, 175]	[150, 176]	[150, 178]
BMI (kg/m^2^)	25.5 ± 4.8	26.5 ± 4.1	27.1 ± 3.4
	[15.6, 34.7]	[15.6, 40.4]	[24.0, 40.4]
Blood glucose (mmol/L)	6.2 ± 1.1	5.7 ± 1.1	5.4 ± 0.9
	[4.8, 8.8]	[3.9, 8.8]	[3.9, 7.2]
Injected activity (MBq)	259.4 ± 54.2	270.1 ± 48.3	268.7 ± 34.8
	[162.8, 376.3]	[162.8, 405.9]	[223.8, 405.9]
Injected activity/weight (MBq/kg)	3.8 ± 0.2	3.8 ± 0.2	3.7 ± 0.2
	[3.5, 4.0]	[3.5, 4.6]	[3.4, 4.6]
Uptake time (min)	72 ± 12	72 ± 15	72 ± 17
	[53, 101]	[50, 107]	[50, 114]
Primary cancer type^*^			
Bone cancer	0/1	0/1	1/1
Breast cancer	2/3	2/3	2/3
Colorectal cancer	1/6	5/6	2/6
Esophagus cancer	0/3	0/3	3/3
Gallbladder carcinoma	0/1	1/1	0/1
Liver cancer	0/2	1/2	1/2
Lung cancer	4/10	9/10	8/10
Lung granulomatous inflammation	0/1	1/1	0/1
Lymphoma	2/12	8/12	6/12
Malignant melanoma	0/1	1/1	1/1
Nasopharynx cancer	0/3	2/3	2/3
Neck cancer	1/1	0/1	0/1
Endometrial	0/1	1/1	1/1
Thyroid cancer	1/1	1/1	0/1
Urothelial carcinoma	0/1	1/1	0/1
Uterine cancer	0/1	1/1	1/1
Total	11/48	34/48	28/48

Study 1 stands for the study of PET image quality, study 2 for the study of sub-centimeter lesions quantification and study 3 for the study of lesions quantification in overweight and obese patient.

*a/b: a is the patient number enrolled in the group; b is the total patient

### PET/CT acquisition and reconstruction

All patients were scanned with a digital PET/CT scanner (uMI 780, United Imaging Healthcare, Shanghai China). The PET scanner sensitivity was 16 kcps/MBq and the spatial resolution was 2.9 mm with a time-of-flight (TOF) resolution of 450 ps. First, the patients underwent a CT scan with a fixed tube voltage of 120 kV and an auto-mAs technique for dose modulation (range 15–100 mA), which provided anatomical information and attenuation correction for the PET images. The PET acquisition range was 5–7 bed positions per patient, according to height, with an overlap of 35%. Then, a PET scan was conducted in step-and-shoot mode. For the visual and quantitative imaging analyses, the PET data were acquired for 3 min/bed and reconstructed with different acquisition times ranging from 0 to 3 min/bed with an interval of 0.5 min/bed. In the studies of sub-centimeter lesion quantification and PET imaging for overweight and obese patients, we performed PET scans for 2.0 min/bed, which was based on the image quantity study below. PET images were reconstructed using two algorithms: OSEM and DPL, respectively. The OSEM algorithm was implemented in 2 iterations, 20 subsets, a Gaussian filter with full width at half maximum of 3 mm, 150 × 150 matrix, 600 mm field of view (FOV), 2.68 mm slice thickness, as well as TOF and resolution modeling. The DPL algorithm was implemented in the same FOV, matrix, and slice thickness as the OSEM algorithm. We also applied standard corrections (scatter, random, dead time, decay, attenuation, and normalization) to both OSEM and DPL reconstructions.

### Training and test of DPL network

DPL network has been established in the previous paper [16], thus, we describe it here briefly. DPL network, which was trained in 2D, was composed of two networks: CNN-DE and CNN-EH. The training dataset was constructed with 161,040 image slice pairs (including 53,680 and 107,360 slice pairs for the 2.4 mm and 1.2 mm, respectively). The test dataset was constructed with 40,260 image slice pairs (including 13,420 and 26,840 slice pairs for the 2.4 mm and 1.2 mm, respectively) from 20 patients were used to construct the test dataset. For CNN-DE neural network, PET images with 10% uniformly down-sampled counts were used as training input. For CNN-EH neural network, PET images with insufficient iterations were used as training input. PET images with full counts and sufficient iterations were used as training targets. The training image size was 249 × 249 × 671 with a voxel size of 2.4 × 2.4 × 2.68 mm^3^. The parameters of DPL network were initialized with Kaiming initialization. We took the loss function as the objective function and used the backpropagation algorithm to update the parameters based on the adaptive moment estimation optimization algorithm and cyclical learning rate. The minimum and maximum values for the cyclical learning rate were 1e–5 and 1e–4, respectively. All the training was performed by using Pytorch 1.5.0 on a computer cluster of 4 × NVIDIA Quadro RTX 6000 GPU. The DPL network is trained on uExploror total-body scanner, and applied to other scanners.

## Statistical analysis

Continuous parameters were presented as the mean ± SD and range. PET images reconstructed by OSEM were used as a reference. All the quantitative parameters were tested for normality using the Kolmogorov–Smirnov test and the two-tailed paired-samples t-test was subsequently performed. Inter-rater reliability was evaluated by using Cohen’s weighted kappa (linear) coefficient. The scores of the qualitative image quality were subsequently compared using the Mann–Whitney U test (Matlab 2020a). Statistical significance was considered when the paired *p* value < 0.05.

## Results

### Visual imaging analysis

**Fig. 1:**
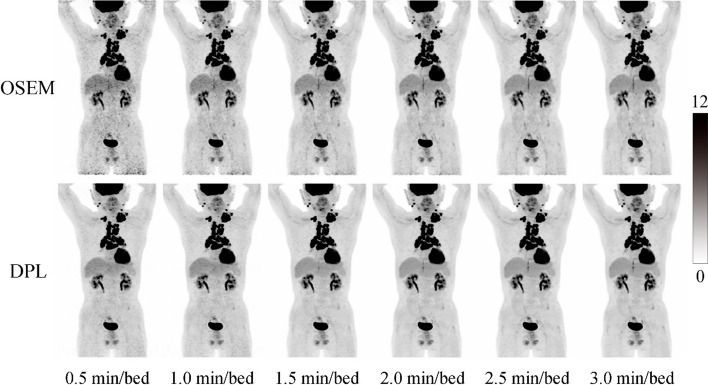
MIP images reconstructed by OSEM and DPL for a patient

**Fig. 2:**
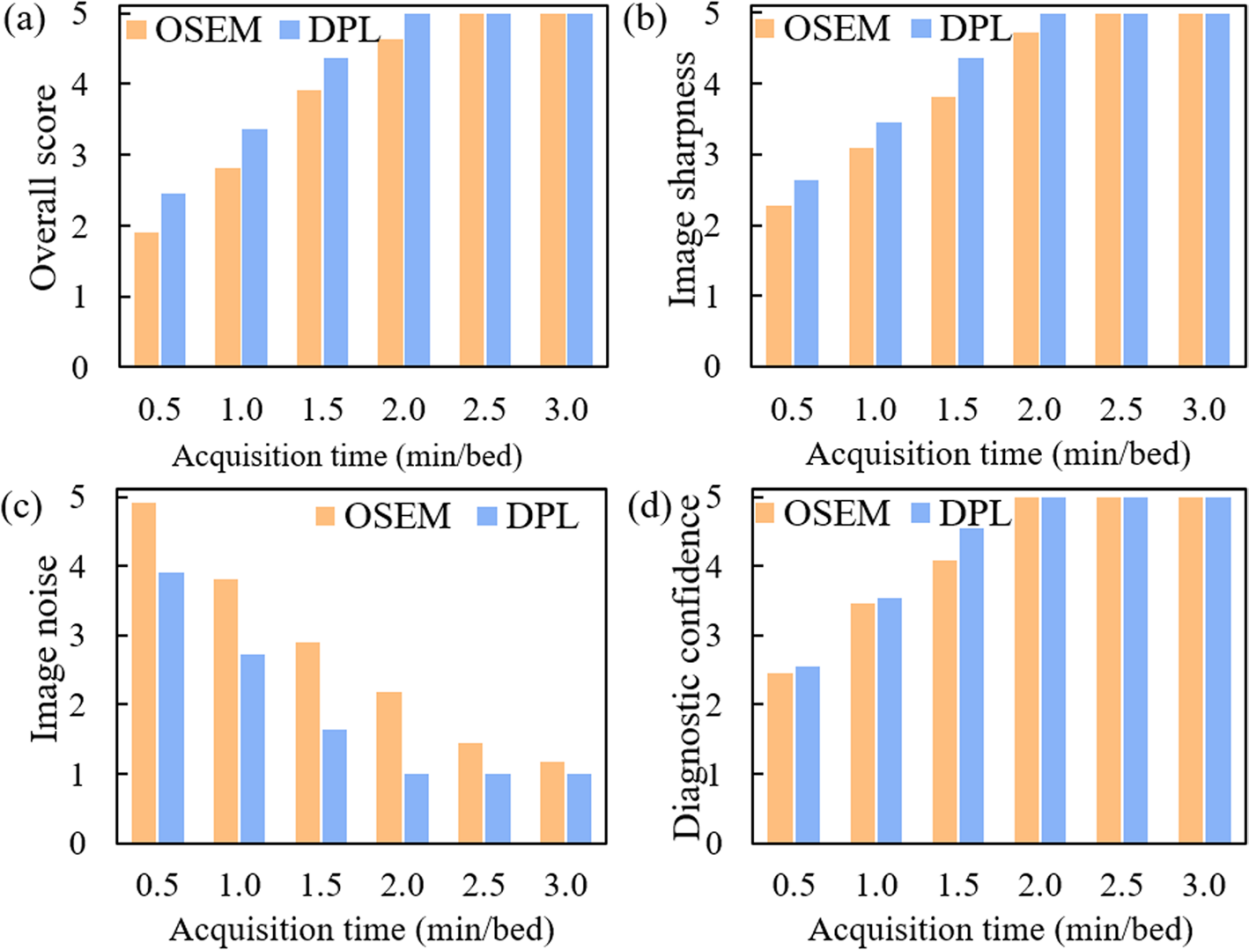
Visual PET image quality of OSEM and DPL varied with acquisition time. **a–d** are overall score, image sharpness, image noise and diagnostic confidence, respectively, which are four criteria used to assess the PET image quality. Each criterion is rated on a 5-point scale, where 1 means poor and 5 means excellent, except for image noise, where 5 means maximum noise and 1 means minimum noise

We compared the image quality and noise levels of OSEM and DPL groups by visual method (Figs. 1, 2) for different acquisition times. Figure 1 shows the MIP images reconstructed from a representative patient at different acquisition times (from 0.5 to 3.0 min/bed). When the acquisition time was less than or equal to 2.0 min/bed, DPL had better image quality than OSEM. However, when the acquisition time exceeded 2.0 min/bed, there was no visual difference between the two algorithms in terms of image quality and noise levels. In addition, when the acquisition time was 0.5, 1.0 and 1.5 min/bed, OSEM resulted in a relatively higher noise level, while DPL maintained a low noise level even at 0.5 min/bed. Figure 2 shows the changes in visual image quality scores (overall score, image sharpness, image noise and diagnostic confidence) with acquisition time for both algorithms. The overall score, image sharpness and diagnostic confidence increased with the acquisition time up to 2.0 min/bed. When the acquisition time exceeded 2.0 min/bed, these scores gradually reached a plateau. The image noise of OSEM reduced as the acquisition time increased from 0 to 3.0 min/bed while the image noise of DPL decreased to a minimum level when the acquisition time was 2.0 min/bed.

### Quantitative imaging analysis

**Fig. 3:**
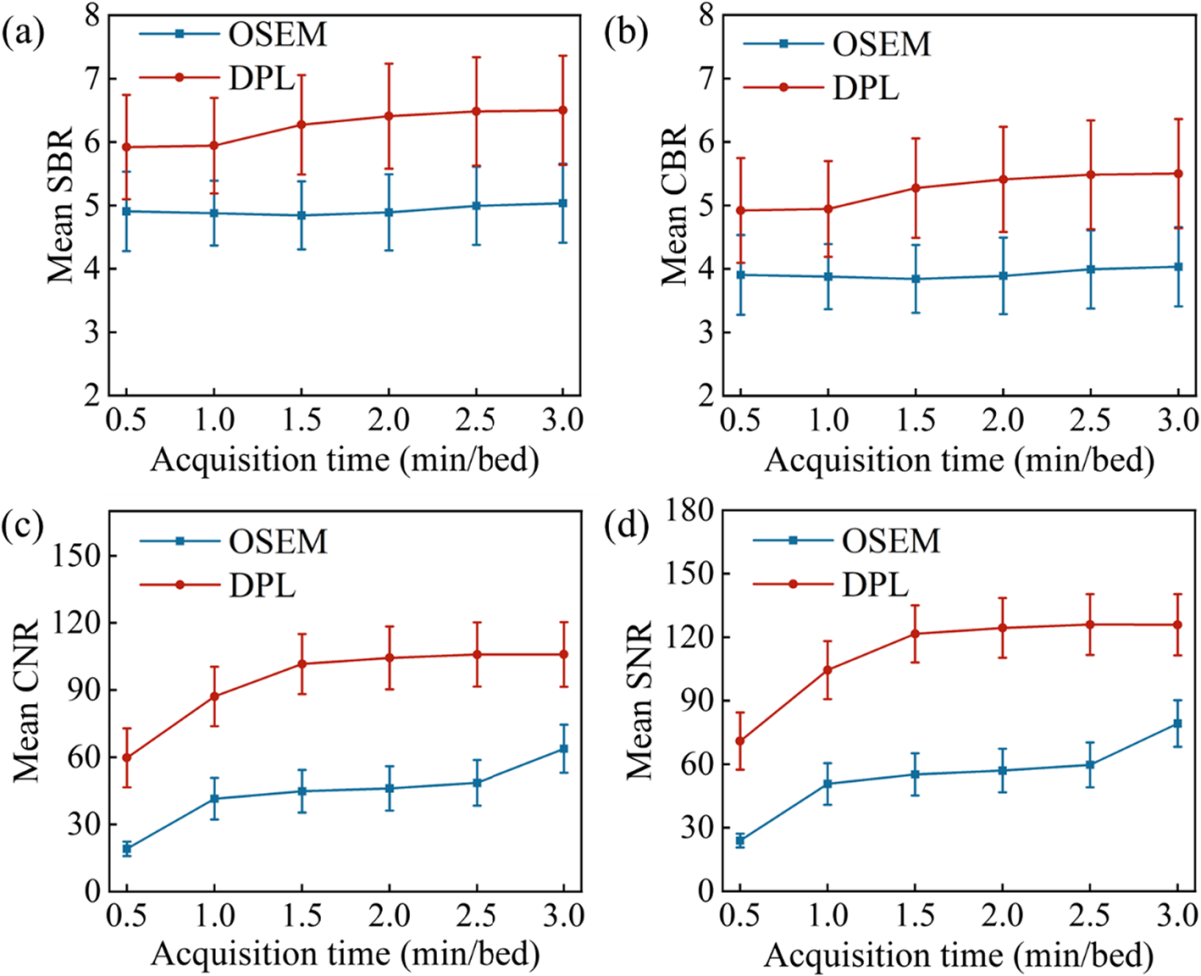
Quantitative analysis of PET image reconstructed by OSEM and DPL algorithms. **a–d** are SBR, CBR, SNR and CNR, respectively, which change with acquisition time. DPL shows a significantly elevated in SBR, CBR, SNR and CNR compared to OSEM (all the paired *p* < 0.001), indicating the improvement of image quality

The values of the SBR, CBR, SNR and CNR of OSEM with DPL for different acquisition times were compared in Fig. 3. On one hand, the SBR and CBR of OSEM were about 5.0 and 4.0, respectively, and did not vary significantly with the acquisition time. On the other hand, the SBR and CBR of DPL were about 6.0 and 5.0, respectively, and increased with the acquisition time up to 2.5 min/bed, after which they remained constant. The SNR and CNR of DPL were significantly higher than those of OSEM for all acquisition times (all the paired *p* < 0 0.001). In addition, the SNR and CNR of DPL also increased with the acquisition time up to 2.5 min/bed, and then stabilized. These results indicated that DPL had better image quality and contrast than OSEM in terms of these quantitative measures.

### Sub-centimeter lesion quantification

**Fig. 4:**
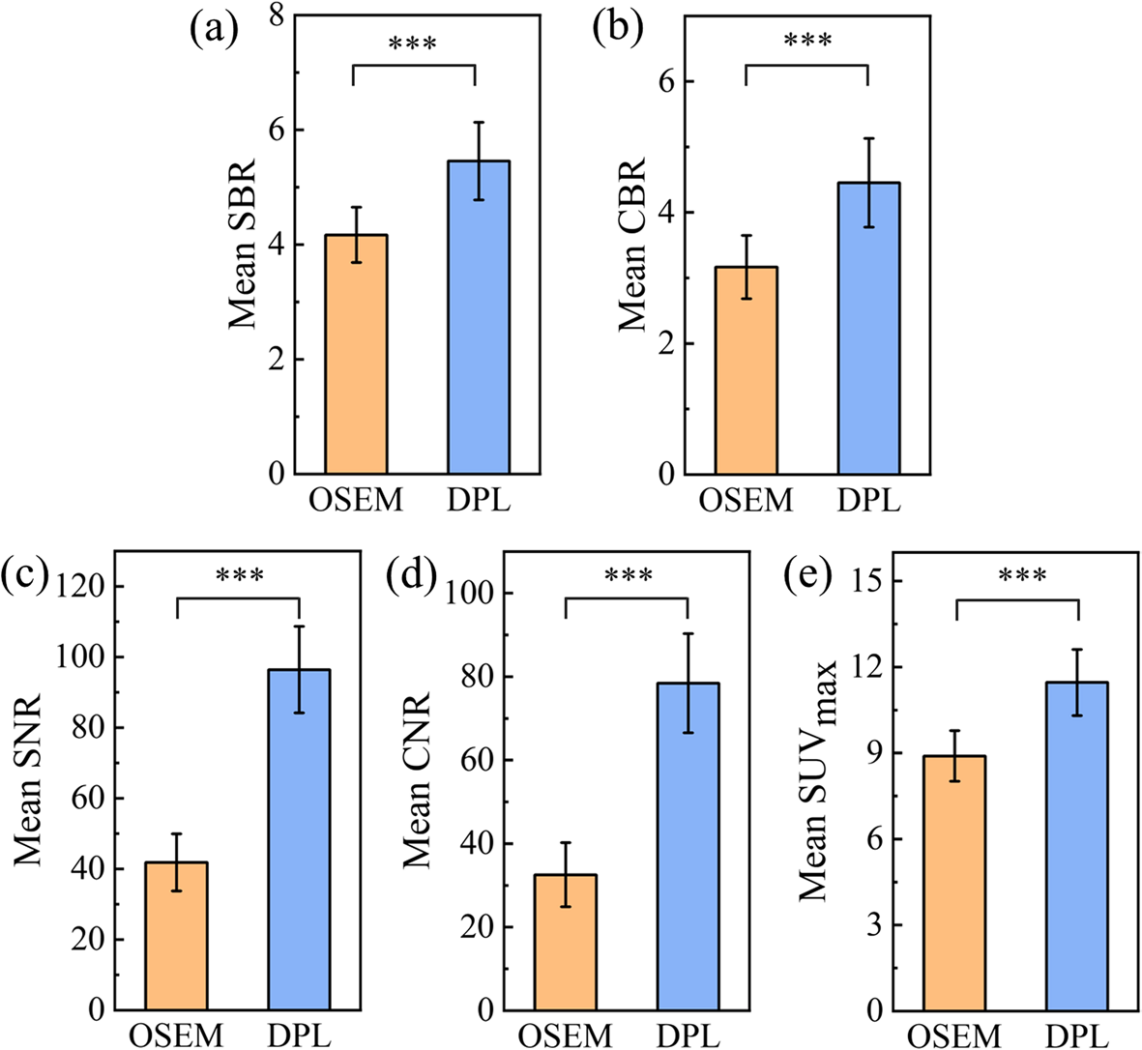
Performances of OSEM and DPL for sub-centimeter lesions quantification. **a–e** are the values of SBR, CBR, SNR, CNR and SUV_max_, respectively. *** means *p* < 0.001

Our study included 63 sub-centimeter lesions with a mean diameter of (0.76 ± 0.15) cm from 34 patients to evaluate the lesion SUV quantification performance of DPL and OSEM. These sub-centimeter lesions encompass four distinct types: lymph nodes (38/63), pulmonary nodules (22/63), liver metastatic lesions (2/63), and rib metastatic lesions (1/63). Figure 4 shows that DPL had significantly higher values of SBR, CBR, SNR, CNR and SUV_max_ than OSEM for these lesions (*p* < 0.001). The SUV_max_ of DPL was 11.46, which was about 30% higher than that of OSEM (8.90). The SBR, CBR, SNR and CNR of DPL increased about 30%, 40%, 130% and 140%, respectively, compared with those of OSEM. These results demonstrate that DPL had better lesion SUV quantification ability than OSEM for sub-centimeter lesions.

### PET imaging for overweight and obese patients

**Fig. 5:**
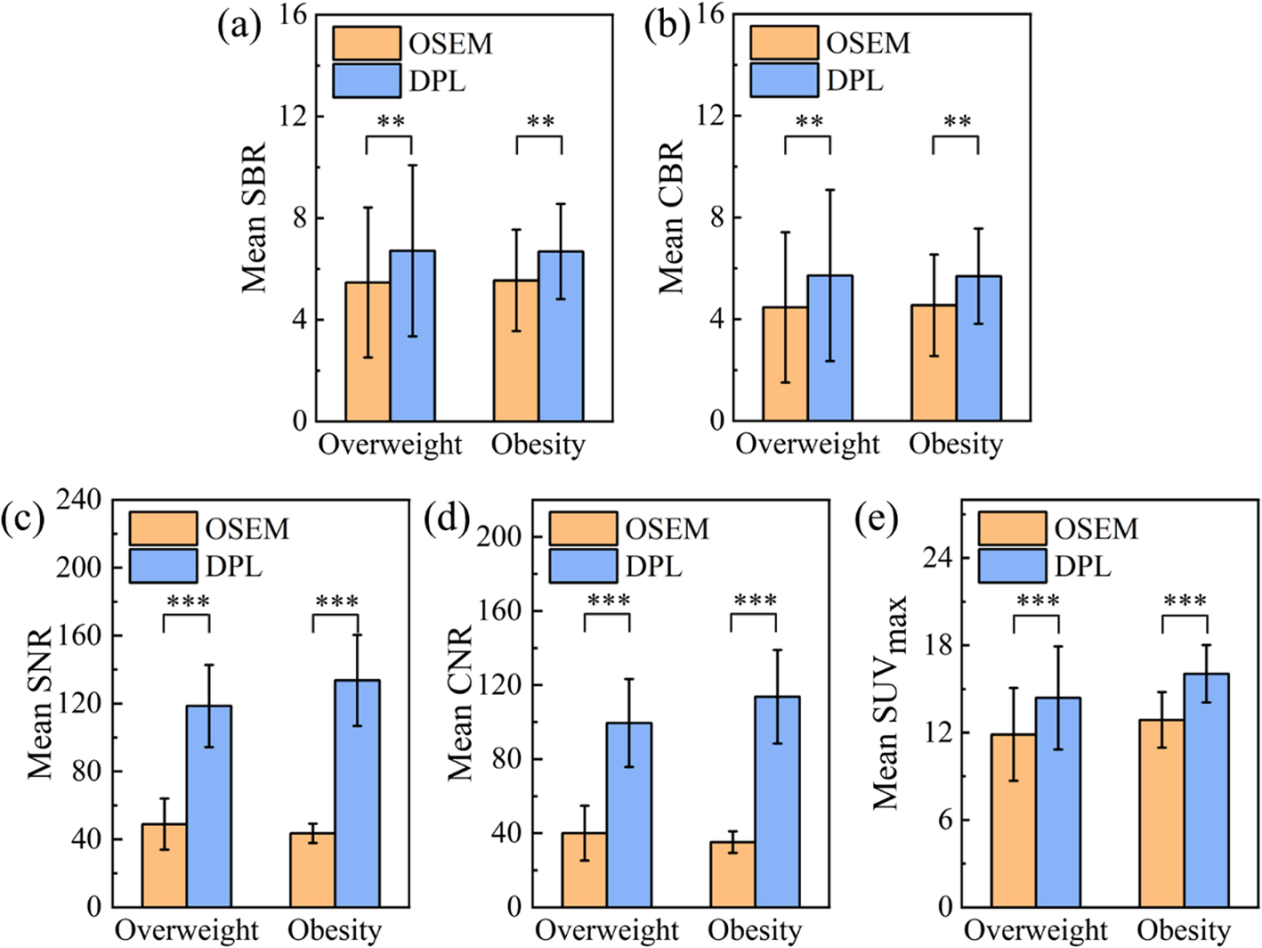
Performances of OSEM and DPL for PET imaging of overweight and obese patients. **a**–**e** are SBR, CBR, SNR, CNR and SUV_max_, respectively. ** means *p* < 0.05 and *** means *p* < 0.001

To evaluate the performance of DPL and OSEM for overweight and obese patients, we enrolled 28 patients with BMIs ranging from 24 to 37.8 kg/m^2^ and 44 lesions (overweight: 20, obesity: 24) in our study. The mean lesion diameter was (1.23 ± 0.42) cm. And, the SUV_max_ of DPL increased by 23% and 24% for overweight and obese patients, respectively, compared with OSEM. The SNR and CNR of DPL were 1.42 and 1.48 times as large as those of OSEM for overweight patients, and more than twice for obese patients (2.07 and 2.23). As shown in Fig. 5, the values of SUV_max_, SBR, CBR, SNR and CNR of DPL were significantly higher than those of OSEM for both overweight and obese patients (*p* < 0.001 for SUV_max_, SNR and CNR; *p* < 0.05 for SBR and CBR). These results indicated that DPL had better lesion SUV quantification ability than OSEM for overweight and obese patients.

### Typical clinical cases

**Fig. 6:**
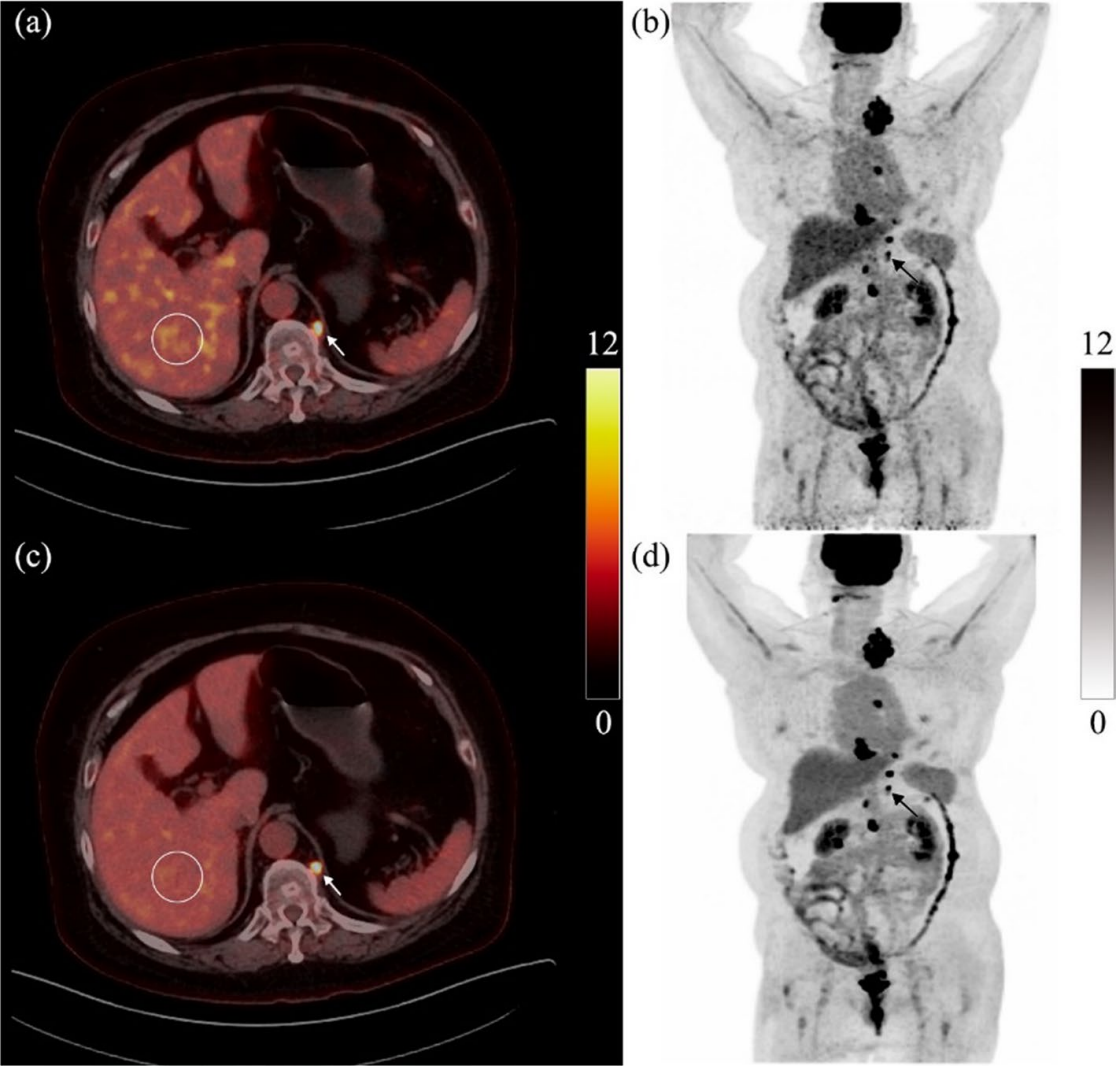
PET/CT image of an obese patient. **a** and **c** are PET/CT fusion images reconstructed by OSEM and DPL, respectively; **b** and **d** are MIPs reconstructed by OSEM and DPL, respectively. The circle on the liver was the VOI used to measure the data with a diameter of 3.0 cm. A metastatic para-aortic lymph node (seen the arrow) of 0.86 cm maximum diameter was detected by both methods. The SUV_max_ of this lymph node was 7.8 for OSEM and 9.0 for DPL

Figure 6 shows a typical PET/CT image of an obese patient (female, 58 years old, endometrial cancer, chemotherapy and radiotherapy, height: 165 cm, weight: 110 kg, blood glucose level: 7.2 mmol/L) who underwent a PET/CT scan 67 min after the injection of 405.89 MBq ^18^F-FDG. The SUV_mean_ and SUV_SD_ of the VOI in Fig. 6 were 3.5 and 0.6 for OSEM, and 3.6 and 0.3 for DPL, respectively. Moreover, a metastatic para-aortic lymph node (seen the arrow in Fig. 6) of 0.86 cm maximum diameter was detected by both methods. The SUV_max_ of this lymph node was 7.8 for OSEM and 9.0 for DPL.

**Fig. 7:**
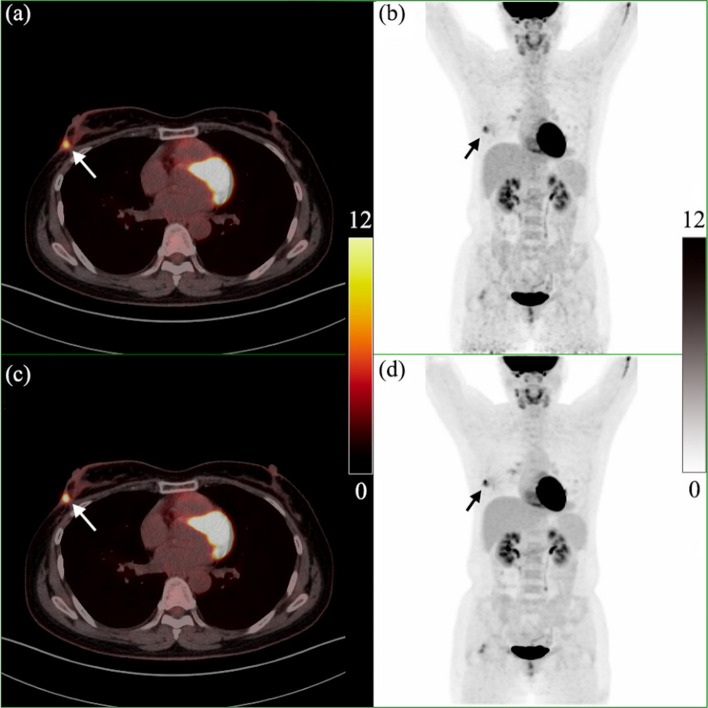
PET/CT image of a breast cancer patient. **a** and **c** are PET/CT fusion images reconstructed by OSEM and DPL, respectively; **b** and **d** are MIPs reconstructed by OSEM and DPL, respectively. A recurrence lesion (seen the arrow) after surgery with a maximal diameter of 0.87 cm was found in the left breast. The SUV_max_ of this lesion was 5.4 for OSEM, and 6.5 for DPL

Figure 7 is the PET/CT image of the second case (female, 50 years old, breast cancer, chemotherapy and radiotherapy after surgery, height: 155 cm, weight: 54 kg, blood glucose level: 5.2 mmol/L). The patient underwent a PET/CT scan 69 min after the injection of 222 MBq ^18^F-FDG. A high-uptake lesion (seen the arrow in Fig. 7) with a maximal diameter of 0.87 cm was found in the left breast, which was proved to be a recurrence lesion by the pathology finally. The SUV_max_ of this lesion was 5.4 for OSEM, and 6.5 for DPL.

**Fig. 8:**
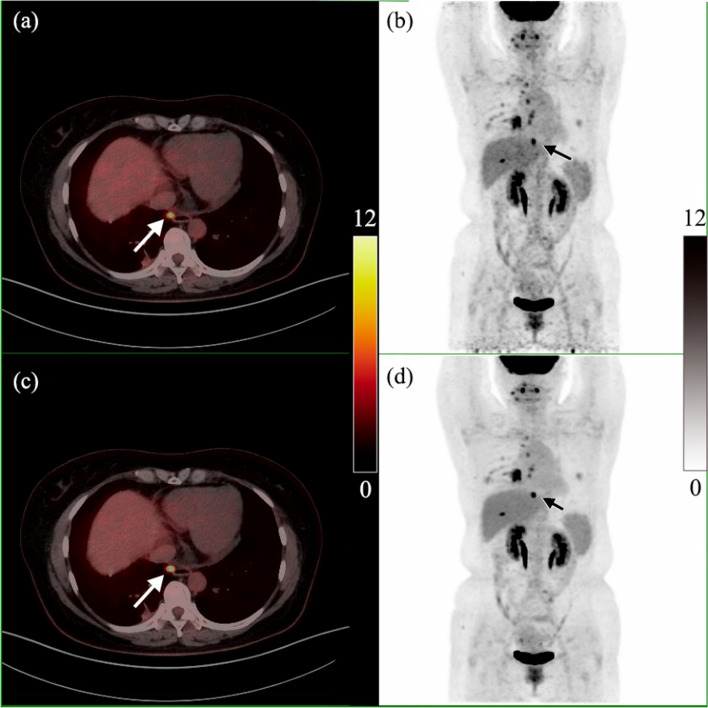
PET/CT image of a patient who was diagnosed with the adenocarcinoma of lung. **a** and **c** are PET/CT fusion images reconstructed by OSEM and DPL, respectively; **b** and **d** are MIPs reconstructed by OSEM and DPL, respectively. The SUV_max_ of the mediastinal lymph node (seen the arrow) with a maximal diameter of 0.67 cm was 6.5 for OSEM, and 8.8 for DPL

Figure 8 is the PET/CT image of a patient (female, 48 years old, lung cancer, targeted therapy, height: 158 cm, weight: 67 kg, blood glucose level: 5.4 mmol/L) who was diagnosed with the adenocarcinoma of lung. The patient underwent a PET/CT scan 64 min after the injection of 246 MBq ^18^F-FDG. Many tumor metastasis lesions were found on the PET/CT images. The SUV_max_ of a mediastinal lymph node (seen the arrow in Fig. 8) with a maximal diameter of 0.67 cm was 6.5 for OSEM, and 8.8 for DPL.

All PET images of three cases reconstructed by DPL had less noises and higher contrasts than those by OSEM.

## Discussion

In this study, we investigated the performance of DPL, a novel deep learning-based PET reconstruction algorithm, on clinical PET/CT images acquired with different acquisition times. We found that DPL improved the image quality and lesion SUV quantification ability compared to OSEM, especially for sub-centimeter lesions in patients and lesions in overweight and obese patients.

In recent years, deep learning methods have demonstrated improved performance in denoting PET images [10, 11]. In this study, we compared PET images reconstructed by OSEM and DPL through visual and quantitative analyses. We found that DPL could improve the image quality and reduce the noise level of PET images when the acquisition time was less than 2.0 min/bed (as shown in Figs. 1, 2). This may lead to higher diagnostic accuracy of DPL-reconstructed images compared to OSEM-reconstructed images. When the acquisition time was equal to or greater than 2.0 min/bed, the visual difference between OSEM and DPL-reconstructed images was not significant (Fig. 1). However, the quantitative analysis of PET images in Fig. 3 showed that DPL still had better quality than OSEM when the acquisition time was between 2.0 and 3.0 min/bed. The reasons for this observation were as follows: First, the SUVs of lesions in DPL-reconstructed images were higher than those in OSEM-reconstructed images, as verified in Figs. 4e and 5e. Second, the SUV_SD_ (representing noise level) measured in OSEM-reconstructed PET images was higher than that in DPL-reconstructed PET images, as evident from a typical PET/CT image of an obese patient, shown in Fig. 6. Third, the fundamental cause of these differences lies in the fact that noise increases with each iteration in OSEM, preventing it from achieving complete convergence. Thus, there was a trade-off between iteration and noise, resulting in partial convergence.

It is worth pointing out that a longer acquisition time does not necessarily translate to better images with high quality. When the acquisition time exceeded or equaled 2.0 min/bed, the SBR, CBR, SNR, and CNR remained stable, as illustrated in Fig. 3. Therefore, based on the visual and quantitative imaging analyses, we recommend an optimal acquisition time of 1.5 to 2.0 min/bed in clinical practice.

Moreover, the quantification of sub-centimeter lesions was crucial for tumor staging, treatment planning, and response monitoring. Some studies have suggested that diagnostic sensitivity decreases when determining the malignancy of small nodules compared to larger ones, and false-negative findings may even occur [20, 21]. However, we observed a significant 29% increase in the SUV_max_ of sub-centimeter lesions when PET/CT images were reconstructed by using DPL, as shown in Fig. 4e. We also found that the SUV_max_ of the sub-centimeter lymph node from an obese patient was enhanced by 15% (7.8 for OSEM and 9.0 for DPL, seen in Fig. 6). The partial volume effect (PVE) is a major factor affecting the SUV accuracy of the sub-centimeter lesion. We selected the lesion SUV_max_ instead of SUV_mean_ because SUV_max_ could be defined maximal uptake of ^18^F-FDG of a voxel. As for the SUV_mean_ and SUV_SD_ of the liver, the average SUV_mean_ and SUV_SD_ are very small. So, the errors of liver SUV_mean_ and SUV_SD_ from PVE can be ignored. In addition, our study suggests that DPL may partially correct the SUV underestimation from PVE and enhance lesion detectability.

Besides, to improve the accuracy of SUV measurements and correct the underestimation of the true SUV, various reconstruction algorithms have been proposed. Wu Z et al. compared the SUV_max_ and SUV_mean_ of 75 small pulmonary nodules that were obtained from different reconstruction methods, including OSEM, OSEM with TOF (OSEM-TOF), OSEM with TOF and point spread function (OSEM-TOF-PSF), and Q.Clear [22]. They found that Q.Clear yielded the highest SUV values for both sub-centimeter and larger nodules, while OSEM-TOF-PSF, OSEM-TOF, and OSEM followed in descending order. However, the PET image quality of Q.Clear was affected by the factors in the penalty function [23], which needed to be carefully adjusted by experience. On the contrary, DPL is a data-driven method which did not require any manual tuning. DPL used two convolutional neural networks (CNN-DE and CNN-EH) to suppress image noise and enhance image contrast respectively. In fact, the TOF resolution of both OSEM and DPL was 450 ps in this study. Since we could not compare DPL and Q.Clear on the same patients due to ethical issues, we only focused on the comparison between DPL and OSEM.

DPL can significantly improve the lesion SUV quantification ability of PET imaging for overweight and obese patients, who usually need a longer acquisition time or a higher injection dose of ^18^F-FDG to obtain satisfactory PET images. As shown in Fig. 6, the PET image reconstructed by DPL had much better noise reduction and lesion contrast enhancement than that reconstructed by OSEM. Furthermore, DPL could also reduce the injection dose of ^18^F-FDG for normal-weight patients while preserving image quality. Wang et al. [17] estimated that DPL could reduce the administered activity of ^18^F-FDG by up to 2/3 in a real-world deployment. Therefore, DPL could play a significant role in reducing radiation exposure, especially for pediatric populations.

However, the proposed DPL method is restricted to tracer-specific. The network must be re-trained using a new dataset or re-tuned via transfer learning for tracers other than ^18^F-FDG. Moreover, more and more novel molecular imaging probes have been developed, which have great potential for precision diagnosis and treatment. In addition, the current implementation of DPL has only two networks (CNN-DE and CNN-EH), which are intended to suppress image noise and enhance image contrast, respectively. We hope that DPL can solve more challenging learning tasks by incorporating more networks in the future.

## Conclusions

The DPL algorithm can achieve significant improvements in PET/CT imaging by reducing the image noise and increasing the lesion SUV_max_. Moreover, DPL is expected to enhance diagnostic confidence with PET/CT imaging, especially for the quantification of sub-centimeter lesions and lesions in overweight and obese patients. These advantages of DPL could lead to better clinical outcomes and patient care.

## Data Availability

Data are available upon request to the corresponding author.

## Abbreviations

BMI: Body mass index
CBR: Contrast-to-background ratio
CNN: Convolutional neural network
CNN-DE: CNN denoising network
CNN-EH: CNN enhancement network
CNR: Contrast-to-noise ratio
COV: Coefficient of variation
DPL: Deep progressive learning
FOV: Field of view
FUSCC: Fudan University Shanghai Cancer Center
GAN: Generative adversarial network
MIP: Maximum intensity projection
OSEM: Ordered subset expectation maximization
PET: Positron emission tomography
PET/CT: Positron emission tomography/computed tomography
PVE: Partial volume effect
SBR: Signal-to-background ratio
SD: Standard deviation
SNR: Signal-to-noise ratio
SUV: Standard uptake value
TOF: Time-of-flight
VOI: Volume of interest
